# Developing a Hypothetical Model for Suicide Progression in Older Adults With Universal, Selective, and Indicated Prevention Strategies

**DOI:** 10.3389/fpsyt.2019.00161

**Published:** 2019-03-26

**Authors:** Tomoe Sakashita, Hirofumi Oyama

**Affiliations:** Department of Social Welfare, Faculty of Health Sciences, Aomori University of Health and Welfare, Aomori, Japan

**Keywords:** suicide, suicide prevention, suicide rate, older adults, Japan, hypothetical model

## Abstract

Suicide prevention is an increasingly important issue, especially among older people. Recent work on improving its effectiveness has focused on developing a framework aligning interventions with key risk factors and stages of the suicide process. We have developed this further, by integrating psycho-behavioral components associated with suicide, existing guidelines for identifying critical points of intervention, and the previous preventive strategies framework. Our schematic diagram shows the relationship between the suicide process and prevention strategies, combined with initiatives for linking different types of strategies, from universal strategies at population level, through selective strategies focusing on groups at risk, to indicated strategies, aimed at specific high-risk individuals. We tested our framework using previous studies assessing the impact of suicide prevention interventions on suicide rates in older adults. It was possible to place all identified interventions within the framework. Examining effectiveness within the framework suggests that some interventions may be more successful in reducing suicide rates because they developed systematic linkages between universal, selective, and indicated prevention interventions. Other studies, however, show that interventions can be successful without these linkages, so other factors may also be important. The main weakness of our framework is a lack of evidence about critical intervention points within the suicide process, which may limit its practical application. However, the framework may help to improve the linkages between types of interventions, and support practitioners in developing a wide range of strategies across different areas and stages of the suicide process.

## Introduction

Suicide is an important public health issue around the world, particularly among older people (those aged over 60) (1). Both suicide rates (2, 3) and the lethality of suicidal behavior (4, 5) are higher in this age group. Older people are more likely to have serious intent to commit suicide, with less warning, than younger people (6, 7).

Several risk factors are associated with suicide among older adults. At least one major psychiatric diagnosis is found in over 70% of suicides in this age group (8). Depression is particularly associated (8), and has a population-attributed risk of more than 40% for suicide, attempted suicide, and suicidal ideation (9–11). Several physical and psychosocial risk factors are also associated, including physical illness and functional impairment (12, 13), and age-related psychosocial stressors, such as lack of supportive social networks (14), loneliness (15), and loss of an important intimate relationship or social role (16).

There are several models of suicidal behavior (17–20). For example, the stress-diathesis theory (17) proposes that longitudinal factors predisposing individuals toward suicidal behavior are influenced by particular stressors. The psychological mechanisms underlying this model remain unclear, however, so it cannot distinguish those at imminent risk of suicidal behavior.

Suicide prevention work has used two basic approaches. The traditional approach is staged: primary, secondary, and tertiary prevention. Primary prevention aims to prevent onset of mental illness, secondary to detect and treat illness, and tertiary to reduce relapses and deterioration (21). The second approach focuses on effectiveness of interventions (2, 17, 22–24). A framework has been created to identify effective interventions, align them with suicidal risk factors, and classify them into three types of prevention strategies, universal, selective, and indicated (25, 26). Universal prevention strategies are applied across populations and individuals not necessarily identified as at particular risk of suicide (25). Selective prevention strategies are aimed at groups at risk of suicide, but not necessarily showing suicidal behavior. Indicated prevention strategies focus on high-risk individuals, such as those who have previously attempted suicide.

Suicide risk at individual level fluctuates over time (18), so efforts to reduce mortality from suicide among older adults in the community need to work with those at various levels of risk. The use of universal, selective and indicated strategies can both address stage of illness, and consider target populations (21), making it more suitable than the staged approach. Research suggests that the risk of suicidal acts could be reduced through a multilevel approach, linking different types of prevention strategies (27). However, no studies have explicitly examined the connections between levels or types of strategy. Another promising model (28) links the suicide process in older adults with the prevention framework (25, 26). It shows that indicated strategies are suitable for individuals with proximal risk factors for suicide (such as depression), selective strategies for those with distal risk factors (e.g., stress or illness), and universal strategies for the entire population, irrespective of risk status. The authors did not, however, show precisely how interventions addressed particular risk factors. This paper therefore aimed to further develop the framework to bring together the suicide process and prevention strategies at different levels.

It is important to ensure that individuals at higher risk of suicide participate in universal or selective interventions, so that they can be identified and supported appropriately. Making better, more systematic linkages between different types of prevention strategies may help with this. However, multilevel interventions and the linkages between them may have different effects (29). The effects may also vary in different age and population groups (30). This paper therefore draws on studies assessing the impact of suicide prevention interventions on suicide outcomes in older adults. It examines whether there were systematic linkages between universal, selective and indicated prevention interventions in studies evaluating the impact of interventions on suicide risk.

## Methods

### Hypothetical Schema of Suicide Progression With Universal, Selective, and Indicated Prevention Strategies

This study built on previous work to develop a framework for suicide prevention, including the steps of the suicide process and time points for specific interventions (31). We combined this with the universal, selective, and indicated preventive strategies framework (25, 26), and models of suicidal behavior (28, 32) to generate a schematic diagram of the suicide process and classify prevention activity by stage.

**Figure 1:**
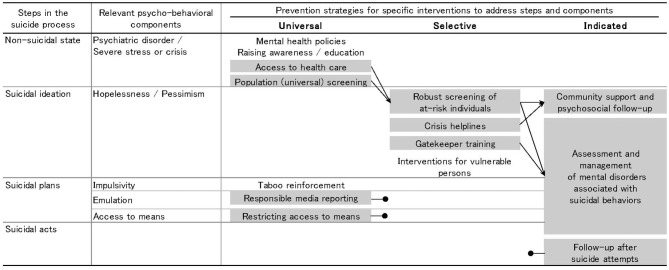
Schematic diagram of suicide process and prevention strategies (29). Interventions highlighted in gray are supported by evidence of their efficacy in reducing suicide risk. A black arrow indicates a clear link to another intervention. A black circle indicates no known link to other interventions. The interventions at each point in the suicide process are expected to involve people at stages closer to suicide.

Figure 1 shows the schema for suicide prevention (29). The left shows the four sequential steps of the suicide process: a non-suicidal state, suicidal ideation, suicidal plans, and suicidal acts. The right shows preventive strategies classified by suicide process stage and type of intervention (2, 23).

The second column integrates the stress-diathesis model for suicidal behavior (32). This model identifies particular psycho-behavioral components that may lead to progression to the next step. For example, depression, hopelessness, suicidal ideation, and impulsivity are suicide risk factors for all age groups, although acute deterioration and acute psychosocial crises are the most important predictors of suicidal ideation. Similarly, hopelessness and pessimism can lead to suicidal thoughts and plans. Emulation and access to means are also important factors in suicide (33, 34). Certain interventions may act on particular psycho-behavioral components, so can minimize progression to the next stage (31). Conwell et al.'s (28) risk factors overlap significantly with the stress-diathesis model. It is therefore helpful to link the elements of the stress-diathesis model to universal, selective, and indicated prevention strategies.

### Testing the Framework Against Evidence on Suicide Prevention Programs

We wanted to know whether the schematic diagram explained findings about suicide prevention interventions. We used a literature search to identify systematic reviews and reports of systematic reviews as reliable sources of articles on intervention types and linkages (see Appendix 1). We included studies assessing the impact of suicide prevention interventions on suicide rates in older adults, particularly initiatives linking different types of prevention strategies.

We examined all the interventions against the diagram. We used previous studies (2, 17, 24) to categorize interventions within universal, selected and indicated strategies.

## Results

### Identifying Studies to Test the Diagram

We identified 53 review articles, and excluded 46 because they did not meet the criteria (Appendix 1). One article (23) was added following cross-referencing. One report (2) was substituted for an overview (35). We found four systematic reviews (24, 36–38), two overviews (29, 39) and two reports of systematic reviews (2, 23), and their reference lists gave 17 suitable studies to test the hypothetical schema and six to examine the effect of linkages between levels of intervention on suicide rate at the population level.

### Universal Prevention Strategies

Universal prevention strategies included mental health policies, awareness-raising and education, improving access to health care, and population or universal screening.

#### Mental Health Policies, Raising Awareness, and Education

Combining these interventions may prevent individuals from developing suicidal ideation (31). However, several systematic reviews have indicated that these interventions alone do not reduce suicidal activities among adults (40, 41). They may facilitate other preventive interventions that address acute deteriorations and psychosocial crises (42, 43), but there is no evidence that they are usually designed to be integrated directly with these strategies.

#### Population Screening for Mental Illness

Access to healthcare (2) and universal screening (29) have both been linked to selective preventive strategies, often because they involve the same systems and staff. One study found that community-based interventions, involving universal, in-depth screening and general care for all older people in a region with a high suicide rate, resulted in a lower suicide rate (44). These community-based interventions used a self-report instrument to assess the risk of depression among older people, and therefore identify those who may otherwise not seek help. This first stage was linked to a selective intervention involving in-depth screening of those identified as at risk, for example, because they were depressed, which is a known suicide risk factor (9–11). This provided a systematic link from universal to selective prevention. Improving education and awareness, and incorporating interventions locally, might improve uptake of screening.

#### Responsible Media Reporting and Restricting Access to Means of Suicide

Other universal prevention strategies include taboo reinforcement to minimize impulsivity, responsible media reporting to minimize emulation, and restricting access to means of suicide. There is evidence that some of these interventions can reduce suicide rates (2, 23, 24, 36), but no proof for others, notably taboo reinforcement. Responsible media reporting and restricting access to means are both supported by evidence, in one case of short-term benefits and in the other of benefits limited to the specific means of suicide (17). These interventions may reduce the frequency of progression toward suicidal acts (31), but are hard to integrate with selective or indicated strategies because they do not allow selection or identification of individuals to target further intervention.

### Selective Prevention Strategies

Selective prevention interventions included gatekeeper training for physicians, robust screening, and counseling of at-risk individuals, the availability of crisis helplines, and interventions for vulnerable people (e.g., those experiencing severe stress).

#### Identifying and Referring At-Risk Individuals

Studies have found that gatekeeper training for physicians to enable them to detect and treat depression can reduce suicide rates (45, 46), probably because depression is such a common risk factor for suicide in this age group (9–11). This, like robust screening and counseling, integrates universal, and selective strategies. Previous community-based interventions using this combination resulted in lower suicide rates. These interventions involved universal, in-depth screening of older people in a region with a high suicide rate. Those who were depressed or suffering from depressive episodes were referred for semi-structured clinical interviews (44). These interventions may help to minimize hopelessness and pessimism, and therefore stop progression toward suicidal plans and acts (29, 31). The model suggests that interventions at particular time points might reasonably target people at stages closer to suicide.

#### Crisis Helplines and Telephone Counseling

Crisis helplines and emergency response services also reduce suicide. One study (47) provided regular telephone support for at-risk individuals and an emergency response when required. This resulted in a lower suicide rate among older women. A community agency that provided telephone counseling with emotional support, crisis intervention, referral services, and home visits reduced hopelessness, but not depressive symptoms (48). Crisis helplines, even without subsequent support, can reduce suicide risk among callers during the call itself and over subsequent weeks (49).

These selective strategies are usually closely linked to indicated strategies, often follow-up with specific individuals, but few rigorous studies have evaluated the efficacy of interventions targeted at specific vulnerable groups.

### Indicated Prevention Strategies

These interventions included assessment and management of mental disorders associated with suicidal behaviors, community support, and psychosocial follow-up.

#### Management of Mental Disorders Associated With Suicidal Outcomes

The appropriate management of mental illness can minimize hopelessness, pessimism, and impulsivity, reducing the likelihood of individuals developing suicidal thoughts or taking action (31, 32). Antidepressants (50, 51) and collaborative care (52, 53) of older people with depression have been associated with reduced risk of suicidal ideation in institutional settings. A secondary analysis (50) of data from three studies on late-life major depression found that antidepressants and interpersonal psychotherapy reduced suicidal ideation. Other studies investigated the effect of antidepressants on suicidal ideation and behavior in patients with late-life major depression (51) and the 2-year effect of collaborative primary care interventions on suicidal ideation and depressive symptoms among older people (52). Interventions included antidepressant treatment, patient education, interpersonal psychotherapy, and care management. Both studies (51, 52) found reduced suicidal ideation among the target group. Collaborative depression care can also reduce suicidal ideation among older people (53). The sample size of treatment studies in institutional settings is usually too small to detect changes in suicide rates, but the link to risk factors such as depression (9–11) suggests that these interventions may be clinically useful to reduce suicidal acts.

These studies suggest that appropriate management of mental illness is an important part of suicide prevention, and can reduce the risk of progression toward suicide ideation, planning, and attempts. However, it may be possible to make a more direct link. One previous study in northern Japan detected individuals with depression via screening interventions (44) and successfully treated them for depression via psychiatric or primary care. This linked the management of mental illness directly with selective preventive strategies, and fits with our model's suggestion that these interventions may become more effective by linking different levels.

#### Community Support and Psychosocial Follow-Up

Follow-up care after attempted suicide is associated with positive outcomes, such as reduced risk of re-attempting (54–56). Follow-up care among recently discharged patients was effective in reducing suicide attempts and deaths in all age groups (54), especially those discharged from emergency departments (35, 55). This intervention may work by reducing the risk of re-attempting (56) and therefore of death by suicide, but the small numbers mean that population suicide rates are not affected. This care is also not usually designed to be integrated with other prevention strategies (37, 54), which might limit its benefits.

### Linkages Between Types of Intervention

Table 1 shows the main characteristics of recent studies evaluating multilevel programs to reduce suicide rate among older adults, and shows specific linkages between types of intervention. For example, two studies linked selective and indicated interventions by emergency calls for help (47) and treatment and referrals (46). They found lower suicide rates among older women in the intervention group (47) and in the population of the intervention area (46). One study evaluated systematic linkages across types of interventions, such as recommendations to move from universal to selective interventions (44), and found reduced suicide rates in both older men and women.

**Table 1:** The main characteristics of recent studies evaluating multilevel programs to reduce suicide rate among older adults.

**Study**	**Design**	**Target population**	**Intervention type and linkage**					**Outcome**
			**Universal**	**Linkage**	**Selective**	**Linkage**	**Indicated**	
De Leo et al. (47)	Large cohort study comparing with the general population	Older users of service			Regular phone call for assessment and emotional support	Emergency call for help	Contacts with trained staff	Lower suicide rate among users. Significantly fewer suicides in women. No difference in male suicide rate.
Szénto et al. (46)	Large quasi-experimental	Adults of all ages in the community			Improved detection of depression following physician education	Recommendation for treatment and referral	Treatment by physician or psychiatrist	Significant reduction in suicide rate compared with control and trend.
Oyama et al. (44)	Meta-analysis of quasi-experimental studies	Older adults living in the community	Universal screening for depression Public campaign	Recommendation to participate further if screening positive No reported linkage	Robust screening for depression	Referral to physician and other health professionals	Treatment by physician and contact with health professionals	Significant reduction in suicide rate in women. No change in suicide rate in men.
Oyama et al. (44)	Meta-analysis of quasi-experimental studies	Older adults living in the community	Universal screening for depression Public campaign	Recommendation to participate further if screening positive No reported linkage	Robust screening for depression	Referral to psychiatrist or other health professionals	Treatment by psychiatrist or contact with health professionals	Significant reduction in suicide rate in women and men.
Hegerl et al. (57)	Large quasi-experimental	Adults of all ages in the community	Public campaign	No reported linkages but possibly increased patient visits	Improved detection of depression following physician education	Recommendation for treatment	Treatment by physician	Significant reduction in suicidal acts (completed plus attempted suicides) over control.
					Gatekeeper training for community facilitators	No reported outcome of linkage	Community support for suicide attempters with brief intervention	No change in suicide rate.
Székely et al. (58)	Large quasi-experimental	Adults of all ages living in the community	Public campaign (including information on crisis help lines)	No reported linkage but increased participation	Crisis helplines Improved detection of depression following physician education Gatekeeper training for community facilitators	No reported outcome of linkage Recommendation for treatment No reported outcome of linkage	Treatment by physician	Significant reduction in suicide rate compared with control and trend.
Ono et al. (59)	Large quasi-experimental	Adults of all ages living in the community	Public campaign	No reported linkage	Gatekeeper training for community facilitators	No reported outcome of linkage		Significant reduction in suicide attempts in a subgroup of older adults.
					Sporadic visits and screening for at-risk individuals	Referral to health professionals	Community support	No difference in overall change in suicide rates over control.

Three other recent multilevel approaches (57–59) had partial linkages of primary care features (for instance, improved management of depression by physicians) between selective and indicated prevention elements, but no reported linkages between universal and selective elements. These studies reported clear reductions in attempted suicide and death by suicide, but the changes in suicide rate with large sample sizes were less clear.

This suggests that selective and indicated interventions, and close linkages between the two, are more likely to affect suicide among older people. It also suggests that multilevel approaches with systematic linkages between levels of intervention are more likely to affect the suicide rate at the population level than those with partial or subtle linkages between universal and selective interventions, in particular.

## Discussion

Our schematic diagram/framework integrates the stress-diathesis model (32), existing guidelines for identifying the critical time for interventions (31), and Gordon's preventive strategies framework (25). It therefore shows the relationship between the suicide process and suitable prevention strategies (29). Our model builds on that of Conwell et al. (28) by illustrating types of intervention strategy (2), possible linkages between interventions, and interventions related to risk factors from the stress-diathesis model (32). These risk factors are similar to those identified by Conwell et al. but focus on specific interventions rather than precise level of risk.

### Practical Application of the Diagram

We hope that the diagram provides a framework to help practitioners and policy-makers to combine elements of intervention programs at different levels and produce more opportunities for effective intervention. This may, in particular, improve the detection of at-risk individuals, altering their progress through the suicide process, and resulting in fewer suicides.

#### Linking Interventions Across Preventive Levels

The model suggests that one reason why particular interventions may affect suicide rates may be whether the intervention is linked to others at different preventive levels. Linking interventions and levels may mean that preventive action can follow individual trajectories toward suicide more closely than separate interventions, and therefore alter these trajectories more effectively (29). For example, population-based studies of depression screening strategies and intervention among older individuals made a systematic link between universal, selective, and indicated prevention strategies (44). Rigorous community-based studies of interventions promoting regular telephone support and emergency response services among older clients (47), and encouraging physicians to attend gatekeeper training (46), linked selective and indicated strategies. These studies suggest that interventions involving assessment and management of mental illnesses associated with suicidal behaviors may be more effective if integrated with selective strategies targeting at-risk groups.

#### Other Effective Interventions

A number of studies, however, have identified effective interventions that were not linked to other types of prevention strategy. For example, meticulous population-based studies of universal interventions to encourage responsible media reporting and restricting access to suicidal means (17, 24) show that these interventions were not designed to be integrated with other strategies. Hospital-based studies of indicated prevention interventions, including follow-up care after suicide attempts (37, 54), suggest these interventions were also not integrated with other prevention strategies. Both these interventions were apparently effective, however, suggesting that other factors are also important in preventing suicide, such as individual risk factors.

### Limitations and Suggestions for Future Research

The diagram's main weakness is the lack of evidence for the precise critical points in the suicide process. Interventions are probably more effective when targeted at particular risk factors in the stress-diathesis model (32), minimizing progression between stages. However, our model does not identify these precise risk factors, which may limit its practical use. Other weaknesses include the small number of studies used in testing. Using literature reviews to identify studies was convenient, and ensured that only validated studies were examined, but may have limited the number of studies available. Future researchers may wish to use a wider search strategy and include more studies.

## Conclusions

Studies suggest that community interventions are important in reducing suicide in older adults, and that integrating universal, selective, and indicated prevention strategies may be crucial in this process. The most important relationship is probably between selective and indicated prevention interventions. These interventions are tailored to individual risk profiles, targeting those most at risk—for example, because they show signs of depression (9–11). They therefore make best use of scarce resources. Our diagram visualizes the relationships, and can help to ensure that strategies and resources are appropriately targeted and interlinked. We hope that the framework may help to improve the linkages between types of interventions, and support practitioners in developing a wide range of strategies across different areas and stages of the suicide process. It may also help policy-makers to take a more strategic approach to suicide prevention at a population level.

## Author Contributions

TS and HO contributed equally to the literature review and manuscript preparation, including discussion.

### Conflict of Interest Statement

The authors declare that the research was conducted in the absence of any commercial or financial relationships that could be construed as a potential conflict of interest. The reviewer DH and handling editor declared their shared affiliation.
