# Supplementary material for: Developing a Hypothetical Model for Suicide Progression in Older Adults With Universal, Selective, and Indicated Prevention Strategies
Source: Front Psychiatry. 2019 Mar 26;10:161. doi: 10.3389/fpsyt.2019.00161 (PMC6445050; doi:10.3389/fpsyt.2019.00161)
Supplement: Supplementary file 1 [file Data_Sheet_1.docx]

Appendix 1. Search strategy used to identify suitable studies for inclusion

1. We carried out PubMed and PsycINFO searches covering the period from January 2011 to December 2016. The search terms used were *suicide*, *prevention*, (*intervention* OR *interventions*), (*population* OR *elderly* OR *older*), AND (*rate* OR *rates*), with cross reference to specific agents covered in this article. Function limits were used to identify reviews.
2. Studies were excluded where (i) subjects were limited to young and middle-aged people (e.g., students, soldiers) or patients treated solely with a specific pharmacotherapy or psychotherapy (as an indicated prevention strategy), (ii) objectives were limited to risk identification and descriptive statistics, and (iii) articles were not published in English.
3. These selection criteria allowed us to identify systematic reviews and reports of systematic reviews, as a convenient way of identifying all studies on a particular topic. From the reference lists of these articles, we selected studies that evaluated the impact of a multilevel approach on suicide outcomes (suicidal ideation, attempted suicide and death by suicide) and comparative articles on single-level approaches. This enabled us to expand our theoretical model to match recent findings.
4. Finally, studies that evaluated the impact of a multilevel approach on the suicide rate were chosen from the reference lists of the reviews to verify whether the linkage between interventions and their impact existed in the multilevel approaches.
